# Grass leaf structural and stomatal trait responses to climate gradients assessed over the 20th century and across the Great Plains, USA

**DOI:** 10.1093/aobpla/plae055

**Published:** 2024-09-26

**Authors:** Ryan C Donnelly, Jesse B Nippert, Emily R Wedel, Carolyn J Ferguson

**Affiliations:** Division of Biology, Kansas State University – 116 Ackert Hall, Manhattan, KS 66506, USA; Division of Biology, Kansas State University – 116 Ackert Hall, Manhattan, KS 66506, USA; Division of Biology, Kansas State University – 116 Ackert Hall, Manhattan, KS 66506, USA; Division of Biology, Kansas State University – 116 Ackert Hall, Manhattan, KS 66506, USA

**Keywords:** plant traits, climate change, grasslands, herbarium, leaf traits, stomata, precipitation gradient, temperature gradient, switchgrass, Scribner’s panicgrass

## Abstract

**Abstract**. Using herbarium specimens spanning 133 years and field-collected measurements, we assessed intraspecific trait (leaf structural and stomatal) variability from grass species in the Great Plains of North America. We focused on two widespread, closely related grasses from the tribe Paniceae: *Dichanthelium oligosanthes* subsp. *scribnerianum* (C_3_) and *Panicum virgatum* (C_4_). Thirty-one specimens per taxon were sampled from local herbaria from the years 1887 to 2013 to assess trait responses across time to changes in atmospheric [CO_2_] and growing season precipitation and temperature. In 2021 and 2022, the species were measured from eight grasslands sites to explore how traits vary spatially across natural continental precipitation and temperature gradients.

Δ^13^C increased with atmospheric [CO_2_] for *D*. *oligosanthes* but decreased for *P*. *virgatum*, likely linked to increases in precipitation in the study region over the past century. Notably, this is the first record of decreasing Δ^13^C over time for a C_4_ species illustrating ^13^C linkages to climate. As atmospheric [CO_2_] increased, C:N increased and δ^15^N decreased for both species and %N decreased for *D*. *oligosanthes*. Across a large precipitation gradient, *D*. *oligosanthes* leaf traits were more responsive to changes in precipitation than those of *P*. *virgatum*. In contrast, only two traits of *P*. *virgatum* responded to increases in temperature across a gradient: specific leaf area (increase) and leaf dry matter content (decrease). The only shared significant trend between species was increased C:N with precipitation. Our work demonstrates that these closely related grass species with different photosynthetic pathways exhibited various trait responses across temporal and spatial scales, illustrating the key role of scale of inquiry for forecasting leaf trait responses to future environmental change.

## Teaser

We measured how leaf traits of two grass species have changed over a 133-year time period and across the Great Plains of North America. Our results show that aspects of the leaf structure of both species responded over time to increases in atmospheric CO_2_ concentration and precipitation. Across the Great Plains, we found that leaf structure tended to respond more to differences in precipitation than temperature. However, we found that some traits, such as stomatal traits, did not respond as expected to environmental changes, contrasting results from other studies. These results demonstrate the importance of documenting trait–climate relationships across space and time, which can aid in forecasting plant responses to future environmental change.

## Introduction

Plant traits are used to predict species responses to changing environmental conditions including human-induced climate change (Violle *et al.* 2007; Parmesan and Hanley 2015), shifts in nutrient cycling (Bouwman *et al.* 2009), and habitat loss (Helm *et al.* 2005). The responses of species to environmental change across space and time have consequences for understanding changes to individual water-use strategies (e.g. Voltas *et al.* 2015; Carlson *et al.* 2016; Welles and Funk 2021), plant community composition (e.g. Jiménez *et al.* 2011; Cleland *et al.* 2013; Griffin-Nolan *et al.* 2019), and ecosystem-level nutrient dynamics (e.g. De Graaff *et al.* 2006; Campbell *et al.* 2009). These shifts are most commonly assessed by comparing traits across species to understand how environmental change drives shifts in community composition and ecosystem function. However, changes in the environment also impact within-species trait variation (Reich 2014), and facilitate the existence of some species across large environmental gradients (Bachle *et al.* 2018). Thus, intraspecific trait variation is a key determinant in forecasting responses to future environmental conditions (Violle *et al.* 2012), including existing spatial variation and assessments of trait responses over time (variation driven by plasticity and/or adaptation).

Since the Industrial Revolution, atmospheric CO_2_ concentrations have increased from anthropogenic fossil fuel emissions, from around 285 parts per million (ppm) since the year 1850 (McCarroll and Loader 2004) to over 420 ppm as of May 2022 (Keeling *et al.* 2005). Increased atmospheric [CO_2_] increases plant growth and alters plant nutrient concentrations and water-use strategies (Ainsworth and Long 2005). One major response has been the increased ratio of carbon (C) to nitrogen (N) in plant tissues over time (Peñuelas and Matamala 1990; McLauchlan *et al.* 2010, 2017; Brookshire *et al.* 2020; Peñuelas *et al.* 2020). All else equal, as atmospheric [CO_2_] has become more readily available, plants proportionally acquire more C than other elements, such as N. This proportional stoichiometric decrease of nutrients in plant biomass has broad implications for global C and N cycling (Reich *et al.* 2006). As low-quality (high C:N) plant litter becomes available for decomposition by microorganisms, decomposition may slow and lead to increased immobilization or decreased rates of N mineralization, which ultimately can feed back to decrease future available N for plants (Reich *et al.* 2006; Welti *et al.* 2020). Increases in atmospheric [CO_2_] can also decrease soil N availability via progressive N limitation, where elevated rates of photosynthesis retain N in plant biomass (Luo *et al.* 2004).

Plant water-use efficiency (WUE)—the ratio of carbon fixed to water lost via stomata to the atmosphere (Farquhar *et al.* 1989)—tends to increase with increased atmospheric [CO_2_]. WUE is determined by the regulation of the stomatal conductance of a plant over time, coupled with the concentration gradients of CO_2_ inside and outside of the leaf. In general, plant species have been found to have increased WUE when exposed to higher levels of [CO_2_] (Jackson *et al.* 1994; Jianlin *et al.* 2008; Brodribb *et al.* 2009; Haworth *et al.* 2011), though the response may be optimized in angiosperms compared to other lineages, such as ferns and gymnosperms (Brodribb *et al.* 2009). One key indicator of changes in plant WUE over time due to increased anthropogenic CO_2_ is a directional change in the discrimination of ^13^C compared to the lighter ^12^C isotope (Δ^13^C) in plant tissues. Δ^13^C is an independent measurement of temporal changes of δ^13^C in plant tissue over time, which is affected by decreasing levels of ^13^C in atmospheric [CO_2_] due to the burning of fossil fuels (with relatively lower amounts of ^13^C compared to atmospheric [CO_2_]) over the past two centuries (Friedli *et al.* 1986). Analyses of herbarium samples representing the past 200 years have found patterns of Δ^13^C in C_3_ plant tissue decreasing (Peñuelas and Azcón‐Bieto 1992; Pedicino *et al.* 2002), increasing (Zhao *et al.* 2001; Pedicino *et al.* 2002), and unchanging trends (Pedicino *et al.* 2002; del Toro *et al.* 2024). Δ^13^C in C_4_ plants has been found to both increase (Pedicino *et al.* 2002; Eastoe and Toolin 2018; del Toro *et al.* 2024) and remain unchanged (Marino and McElroy 1991; Pedicino *et al.* 2002) over time. These results illustrate that changes in Δ^13^C do not reflect changes in atmospheric [CO_2_] levels; rather, Δ^13^C is linked more tightly to photosynthetic pathway (C_3_ vs. C_4_) or phylogeny (O’Leary 1988; Farquhar *et al.* 1989; Stein *et al.* 2021). In C_3_ plants, the bulk of carbon fractionation occurs during carboxylation by RuBisCO as this enzyme discriminates against the heavier C isotope. δ^13^C in C_4_ plants is less variable given that CO_2_ is concentrated in the bundle sheath, resulting in a higher amount of ^13^C fixation by RuBisCO (Farquhar *et al.* 1989).

Stomatal trait differences including stomatal size, density, and distribution vary among C_3_ and C_4_ grass species, and reflect their evolutionary history (Taylor *et al.* 2012; Zhao *et al.* 2022). Data from herbarium specimens and elevated [CO_2_] chamber studies have revealed that some plant species reduce the number of stomata on their leaves in response to increased [CO_2_] (Peñuelas and Matamala 1990; Beerling and Chaloner 1993a, and 1993b; Knapp *et al.* 1994; Woodward and Kelly 1995; Bettarini *et al.* 1998; Doheny-Adams *et al.* 2012; Large *et al.* 2017). Guard cell length (stomatal size) may also decrease (Miglietta and Raschi 1993). With higher [CO_2_], plants can reduce their stomatal densities to reduce water loss while maintaining similar photosynthetic production. However, this response is not uniform across all species; a wide range of species across different plant families have shown both increases or no changes in stomatal density with increases in [CO_2_] (Beerling *et al.* 1992; Bettarini *et al.* 1998; Ydenberg *et al.* 2021). While herbarium specimens have been used to understand changes in non-stomatal grass leaf traits (McLauchlan *et al.* 2010; Brookshire *et al.* 2020; del Toro *et al.* 2024), we lack a clear understanding of how stomatal traits have changed between the varying photosynthetic pathways over recent centuries.

Many grass species have broad distributions and high abundance across large environmental gradients. Widespread distributions can be partially explained by trait plasticity that underlies tolerance to disparate environmental conditions (Siefert *et al.* 2015; Li *et al.* 2016; Moran *et al.* 2016; Bachle *et al.* 2018). On a broad scale, this may be due to plastic responses to differing environmental factors, such as precipitation, temperature, and soil characteristics (Bernard‐Verdier *et al.* 2012; Westerband *et al.* 2021). Across the North American Great Plains, climate varies substantially due to precipitation and temperature gradients (Kunkel *et al.* 2013; Nielsen 2018), with a cold-to-warm gradient running north to south and a dry-to-wet gradient running west to east. Previous research has shown that for both C_3_ and C_4_ grass species, differences in leaf traits are more often linked to precipitation than temperature gradients, with C_4_ grasses exhibiting significantly more variability than C_3_ grasses (Oyarzabal *et al.* 2008). However, it has not been tested how closely related species with different photosynthetic pathways respond across large environmental gradients. In addition, further insight into how the traits of an individual species respond to differences in precipitation and temperature is necessary to understand how that species may respond to global change.

**Figure 1.**
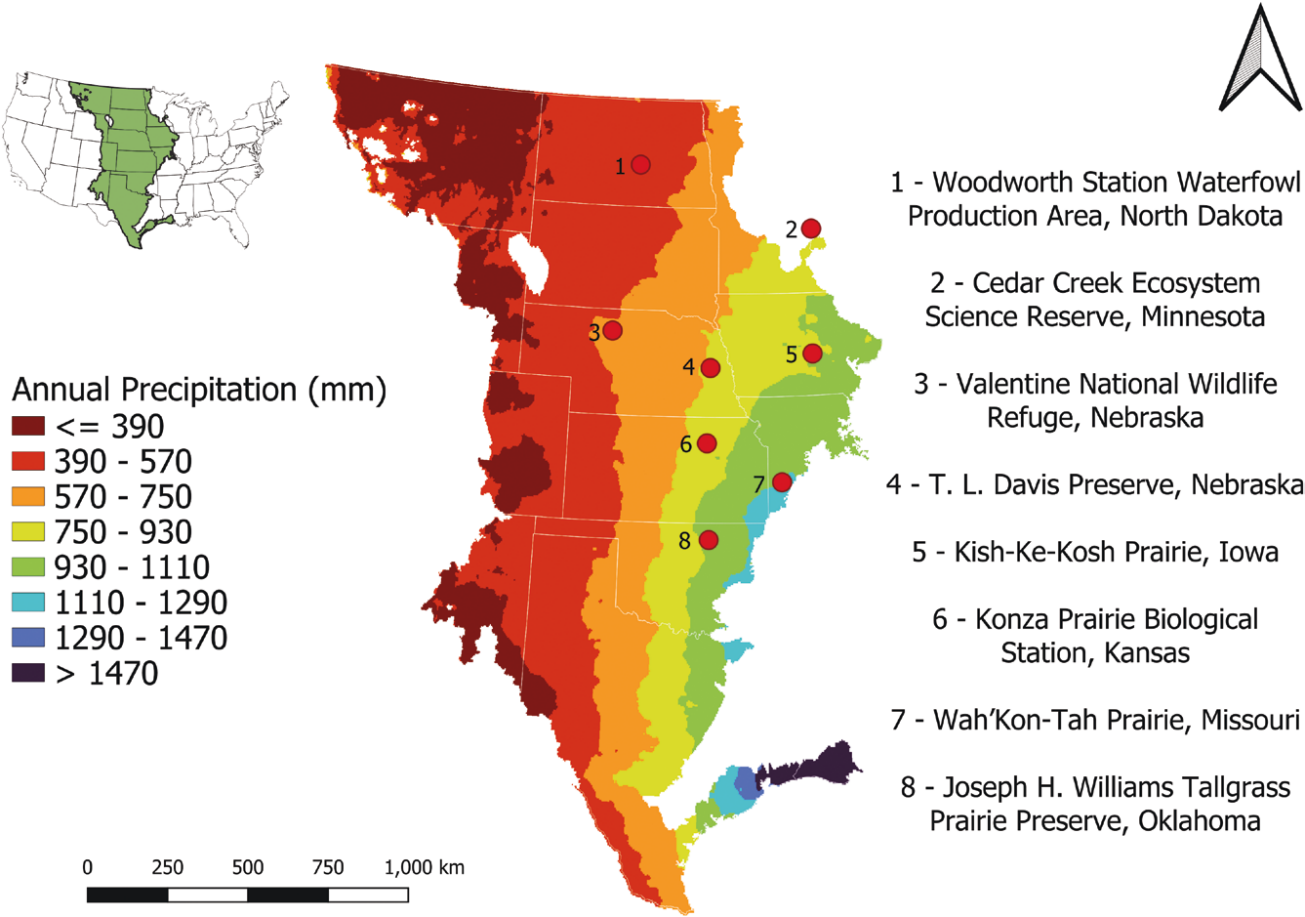
A map of the North American Great Plains ecoregion within the USA and its average annual total precipitation from 1991 to 2020. Each grassland site is represented by a circle and each number corresponds to the site’s name. We used the ecoregion boundary determined by the United States Environmental Protection Agency’s Level I Ecoregions and cropped the boundary to be within the continental USA. Annual precipitation data were retrieved from the PRISM Climate Group at Oregon State University (2022).

To assess temporal and spatial differences among traits of C_3_ and C_4_ grasses, we measured a suite of leaf traits (Table 1) on two closely related (tribe Paniceae) perennial grasses: *Dichanthelium oligosanthes* subsp. *scribnerianum* (Scribner's panicgrass; C_3_) and *Panicum virgatum* (switchgrass; C_4_). These two taxa are common throughout the Great Plains (Great Plains Flora Association 1986) and abundant in local herbarium collections. In this study, we evaluated how functional leaf traits of these two grasses vary over time as atmospheric [CO_2_] has increased by measuring traits from herbarium specimens collected in Kansas. We also assessed intra-taxon variability by measuring traits at eight grassland sites across the Great Plains (Fig. 1). For temporal trends, we predicted Δ^13^C would decrease in *D. oligosanthes* and exhibit no change in *P. virgatum*. *Dichanthelium oligosanthes* is a C_3_ species, which we predict will respond to increased [CO_2_] concentrations by increasing its WUE to either conserve water while maintaining the same rates of photosynthesis or increase photosynthesis and maintain the same rates of water loss, thus decreasing Δ^13^C. We did not expect Δ^13^C of *P. virgatum* to respond over time because discrimination in C_4_ species is minimally affected by [CO_2_] (O’Leary 1988). We also predicted both grasses will increase tissue C:N ratios and decrease stomatal density and stomatal lengths on both sides of the leaves in response to increased [CO_2_] over time. Lastly, we hypothesized %N and δ^15^N would decrease for both taxa as others have found (McLauchlan *et al.* 2010, 2014). Because both taxa are widely distributed across North America and are known to exhibit variation in leaf morphology (Barkworth *et al.* 2003), we expected specific leaf area (SLA) to be greater in areas with warmer temperatures but not be correlated with differences in precipitation (Sandel *et al.* 2021; Griffin-Nolan and Sandel 2023). We expect leaf dry matter content (LDMC) to increase with greater precipitation and decrease with higher temperatures (Ye *et al.* 2024).

**Table 1.** A list of traits measured in this study.

Traits measured across time	Traits measured across space
Total stomatal density (stomata/mm^2^)	Total stomatal density (stomata/mm^2^)
Adaxial stomatal density (stomata/mm^2^)	Adaxial stomatal density (stomata/mm^2^)
Abaxial stomatal density (stomata/mm^2^)	Abaxial stomatal density (stomata/mm^2^)
Adaxial stomatal length (mm)	Adaxial stomatal length (mm)
Abaxial stomatal length (mm)	Abaxial stomatal length (mm)
Stomatal ratio (adaxial:abaxial)	Stomatal ratio (adaxial:abaxial)
Δ^13^C (‰)	δ^13^C (‰)
C:N	C:N
%N	Specific leaf area; SLA (cm^2^ g^-1^)
δ^15^N (‰)	Leaf dry matter content; LDMC
	Leaf thickness (mm)

## Materials and Methods

### Collection of herbarium material and field study sites

To measure temporal trends in leaf traits, we sampled 14 specimens each of *D. oligosanthes* and *P. virgatum* at the Kansas State University Herbarium (KSC) and 17 specimens each at the Ronald L. McGregor Herbarium at the University of Kansas (KANU; **see Supporting Information—Table S1**). KSC boasts a large (ca. 200 000) collection of plant specimens, many of which are historical specimens dating prior to 1900. KANU hosts approximately double (~400 000) the number of plant specimens as KSC, most of which were collected post-1950. Together these herbaria complement each other, allowing us to sample across a wider range of dates (1887–2013) than would have been possible at just one herbarium. For the years 2021 and 2022, plants were collected in the field at Konza Prairie Biological Station, Kansas and pressed and dried before sampling.

We used several criteria to standardize our sampling efforts. First, specimens needed to have ample leafy material, a prerequisite for approval for destructive sampling. Second, all specimens sampled were collected from the eastern third of the state of Kansas to minimize environmental variation by location. Third, all specimens sampled were collected during the species’ respective growing season (May–July for *D*. *oligosanthes* and June–August for *P*. *virgatum*) to avoid senesced material.

To compare how *D. oligosanthes* and *P. virgatum* leaf traits vary across grasslands of the Great Plains of the USA, we sampled individuals from eight sites over the summers of 2021 and 2022 (Fig. 1): (1) Woodworth Station Waterfowl Production Area, North Dakota, (2) Cedar Creek Ecosystem Science Reserve, Minnesota, (3) Valentine National Wildlife Refuge, Nebraska, (4) T. L. Davis Preserve, Nebraska (5) Kish-Ke-Kosh Prairie, Iowa, (6) Konza Prairie Biological Station, Kansas, (7) Wah’Kon-Tah Prairie, Missouri, and (8) Joseph H. Williams Tallgrass Prairie Preserve, Oklahoma. We sampled plants growing from remnant native prairies at all sites except the Woodworth Station Waterfowl Production Area, Cedar Creek Ecosystem Science Reserve, and part of Wah’Kon-Tah Prairie. At the Woodworth Station Waterfowl Production Area, all *P. virgatum* represented restored populations. At Wah’Kon-Tah Prairie, two replicates of *P. virgatum* came from restored populations. Both restored populations were seeded with locally sourced seeds. The restored populations at the Cedar Creek Ecosystem Science Reserve were recovered from the seed bank. *Dichanthelium oligosanthes* was not collected at the Woodworth Station Waterfowl Production Area and *P*. *virgatum* was not collected at Kish-Ke-Kosh Prairie.

### Trait measurements

At each grassland site, one to nine replicates of each species (with most sites having five replicates) were measured for their specific leaf area (SLA), leaf dry matter content (LDMC), leaf thickness, C:N, δ^13^C, stomatal density, and stomatal length using standardized sampling methods (Pérez-Harguindeguy *et al.* 2016). For leaf measurements (SLA, LDMC, and leaf thickness), the most recently produced, but mature leaf was sampled from each replicate. Leaf area and leaf thickness were measured in the field. Leaf area was measured using Leafscan, a mobile app for measuring the surface area of leaves (Anderson and Rosas-Anderson 2017), and leaf thickness using calipers. To calculate LDMC, leaves were rehydrated by being submerged in water for 24–72 hours for wet mass measurements and dried in a drying oven at 60°C for at least 48 hours for dry mass.

Stable isotope measurements for leaf δ^13^C, δ^15^N, total C, and total N were performed at the Stable Isotope Mass Spectrometry Laboratory at Kansas State University. Multiple leaves from each replicate were dried for at least 48 hours at 60 °C and homogenized with an amalgamator. Total C and N of homogenized leaf samples were measured using an Elementar vario Pyro cube coupled to an Elementar Vision mass spectrometer for isotope analysis. Isotopic abundance ratios were converted to *δ* notation using:


$$\begin{array}{l} \delta =\left[\frac{R_{sample}}{R_{standard}}-1\right]*1000 \end{array}$$

where *R* is the ratio of heavy to light isotopes for the sample and standard, respectively. Working laboratory standards were annually calibrated against the internationally accepted standard, Vienna Pee-Dee Belemnite for δ^13^C, and atmospheric air for δ^15^N. Within-run and across-run variability of the laboratory working standard (apple leaves—NIST 1515) was <0.05‰.

For temporal trends, all δ^13^C values were corrected for changes in atmospheric δ^13^C by converting to carbon isotope discrimination values Δ^13^C according to Farquhar *et al.* (1982):


$$\begin{array}{l} \Delta^{13}C=\frac{\delta^{13}C_{air}-\delta^{13}C_{plant}}{1+\delta^{13}C_{plant}/1000} \end{array}$$

Atmospheric [CO_2_] and δ^13^C_air_ measurements were retrieved from McCarroll and Loader (2004) for the years preceding 2004 and measurements from the Mauna Observatory Data were used for years 2004–2022 (Keeling *et al.* 2005).

We measured stomatal density and length using stomatal peels on herbarium samples and pressed and dried field samples collected from each study site. Stomatal peels were created by applying clear nail varnish to leaves of the specimens and peeling the varnish once dry with clear tape. Both *D. oligosanthes* and *P. virgatum* are amphistomatous, so peels were made on both the abaxial and adaxial surfaces of the leaves. For herbarium specimens, the leaves of *P. virgatum* were long and folded to fit on the mounting sheet, exposing both sides of the same leaf. Thus, abaxial and adaxial peels were taken from the same leaf where the leaf was folded. For *D. oligosanthes*, the leaves were short and not folded to fit on the herbarium sheets, so only one side of each leaf was readily available to perform peels. To circumvent this issue, peels of the abaxial and adaxial surfaces were made on different (but similarly developed) leaves of the same individual. For field-collected material, abaxial and adaxial peels were taken from the same leaf.

Two counts of stomatal density were taken for each peel and five replicates of stomatal lengths were measured for each count of stomatal density (10 total per specimen). Stomata were counted under 20× magnification on the objective lens and 10× magnification on the ocular lens using an Olympus BH-2 Microscope (Shinjuku City, Tokyo, Japan). An image was taken of each leaf section using a Lumenera Infinity 2 microscopy camera (Ottawa, Canada). The area of the image field of view was determined by using a stage micrometer and was 0.120 mm^2^ for each image. Stomatal densities were then converted to stomata per 1 mm^2^. Total stomatal density was measured as the sum of the abaxial and adaxial stomatal densities. Stomatal length (horizontal length of the guard cell from end to end) was measuring using ImageJ; pixel length was converted to mm using a reference length determined from the stage micrometer. Five herbarium specimens of *P. virgatum* that were measured for stable isotopes were unable to be sampled for stomatal densities or lengths, as either the specimens had leaves that were too curled or wrinkled to obtain peels, or stomata were too sunken and not visible on the peels. Additionally, we note that because leaves shrink during dehydration, these measurements are likely overestimations of stomatal densities and underestimations of stomatal lengths compared to fresh leaf tissue. However, because all tissue in this study was dry, the values are all comparable.

### Statistical analyses

All statistical analyses were performed in R V4.2.1 (R Core Team 2022). For temporal trait responses, we used linear regression models to determine if traits significantly differed due to changes in environmental variables over time. We performed separate linear regressions for each trait (Table 1) with atmospheric [CO_2_] (ppm) and growing season precipitation (mm) and temperature (°C) as separate predictor variables and month of collection as a random effect to account for natural changes in trait values throughout the growing season. Data for growing season (April—September) total precipitation and average temperature were retrieved from the National Oceanic and Atmospheric Administration’s (NOAA) weather station located in Manhattan, Kansas (Lawrimore *et al.* 2016; Nippert 2019). Historic precipitation and temperature data for years prior to 1891 were not available. For spatial trait responses, we used linear regression models to determine if traits significantly differed due to climactic variation in precipitation and temperature. We performed separate linear regressions for each trait (Table 1) and performed separate models using the mean 30-year growing season precipitation and the mean 30-year growing season temperature as predictor variables that characterize the local climate. These values were retrieved from NOAA’s US Monthly Climate Normals (1991–2020) (Palecki *et al.* 2021) for the closest weather station to each collection site. We determined the length of the growing season for each site separately based on monthly precipitation and temperature. Mean 30-Year Growing Season Precipitation (mm) was calculated by summing the monthly precipitation normal for each month in the growing season for each site and Mean 30-Year Growing Season Temperature (°C) was calculated by averaging the monthly temperature normal for all months in the growing season for each site **[see Supporting Information—Table S2**). All models were performed separately for each taxon.

## Results

### Temporal trends (from herbarium specimens)

**Figure 2.**
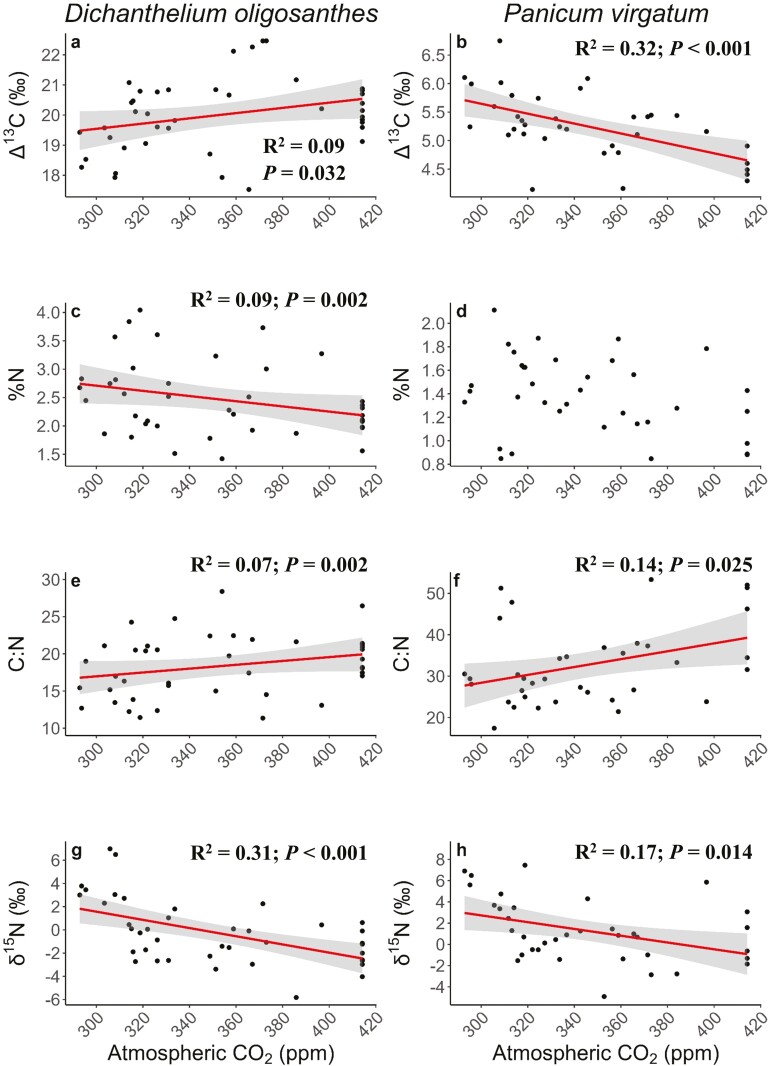
The change in Δ^13^C, %N, C:N, and δ^15^N of *D. oligosanthes* (left column) and *P*. *virgatum* (right column) leaves as atmospheric CO_2_ increased from the years 1887 to 2020. Regression lines and confidence intervals are displayed when *P* < 0.05. Please note differing scales for trait values on the y-axis for both species.

The Δ^13^C of *D. oligosanthes* and *P. virgatum* showed opposite trends as atmospheric [CO_2_] increased over the 20th century. However, the interpretation of these trendlines indicates a similar physiological response—a decrease in WUE over time. The Δ^13^C of *D. oligosanthes* exhibited a significant, positive correlation with atmospheric [CO_2_] (*R*^2^ = 0.09, *P* = 0.032; Fig. 2a), and the Δ^13^C of *P. virgatum* showed a significant, negative correlation with atmospheric [CO_2_] (*R*^2^ = 0.32, *P* < 0.001; Fig. 2b). The %N of *D*. *oligosanthes* exhibited a significant, negative correlation with atmospheric [CO_2_] (*R*^2^ = 0.09, *P* = 0.002; Fig. 2c), decreasing about 20.4% over 133 years. However, %N did not change significantly for *P*. *virgatum* (Fig. 2d). C:N showed significant, positive correlations with atmospheric [CO_2_] for both *D.* oligosanthes (*R*^2^ = 0.07, *P* = 0.002; Fig. 2e) and *P*. *virgatum* (*R*^2^ = 0.14, *P* = 0.025; Fig. 2f). On average, C:N increased about 18.7% for *D*. *oligosanthes* and about 41.6% for *P*. *virgatum* over the 133-year period. Leaf δ^15^N showed significant, negative correlations with atmospheric [CO_2_] for both *D. oligosanthes* (*R*^2^ = 0.31, *P* < 0.001; Fig. 2g) and *P. virgatum* (*R*^2^ = 0.17, *P* = 0.014; Fig. 2h).

**Figure 3.**
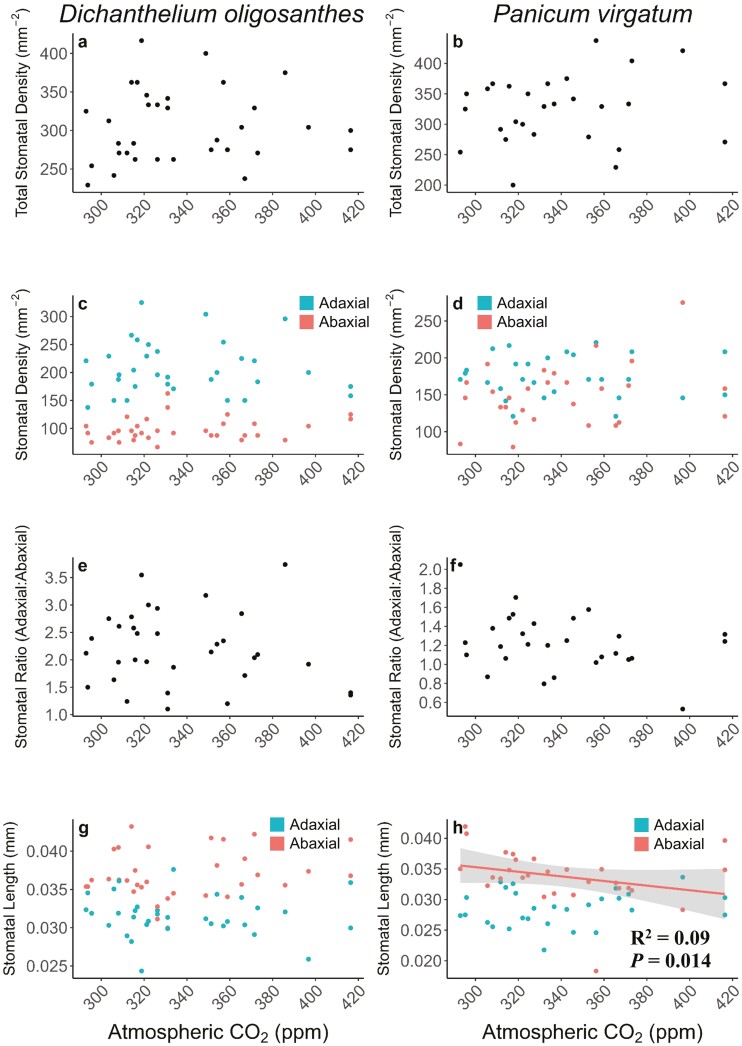
The change in stomatal density, stomatal ratio, and stomatal length of *D*. *oligosanthes* (left column) and *P*. *virgatum* (right column) leaves as atmospheric CO_2_ increased from the years 1887 to 2020. Regression lines and confidence intervals are displayed when *P* < 0.05. Please note differing scales for trait values on the y-axis for both species.

For stomatal traits, the abaxial stomatal length of *P. virgatum* significantly decreased as atmospheric [CO_2_] increased (*R*^2^ = 0.09, *P* = 0.014; Fig. 3h) and increased as temperature increased (*R*^2^ = 0.26, *P* = 0.007; see Supporting Information—**Fig. S1a**). The adaxial stomatal length of *P*. *virgatum* significantly decreased as precipitation increased (*R*^2^ = 0.17, *P* = 0.033; **see Supporting Information—Fig. S1b**) All other stomatal traits for both species were unchanged over time (*P* > 0.05; Fig. 3a–g).

Non-stomatal leaf traits also responded to differences in precipitation or temperature across time. The %N of *D*. *oligosanthes* showed a significant, negative correlation with precipitation (*R*^2^ = 0.18, *P* = 0.008; **see Supporting Information—Fig. S2a**) and a significant, positive correlation with temperature (*R*^2^ = 0.10, *P* = 0.009; **see Supporting Information—Fig. S2b**). C:N exhibited a significant, positive correlation with precipitation for *D*. *oligosanthes* (*R*^2^ = 0.13, *P* = 0.027; **see Supporting Information—Fig. S2c**). Lastly, the Δ^13^C of *P*. *virgatum* showed a significant, negative correlation with temperature (*R*^2^ = 0.19, *P* = 0.021; **see Supporting Information—Fig. S2d**). For both species, leaf δ^15^N did not respond to differences in precipitation or temperature across time.

### Spatial trends (across grassland sites)

**Figure 4.**
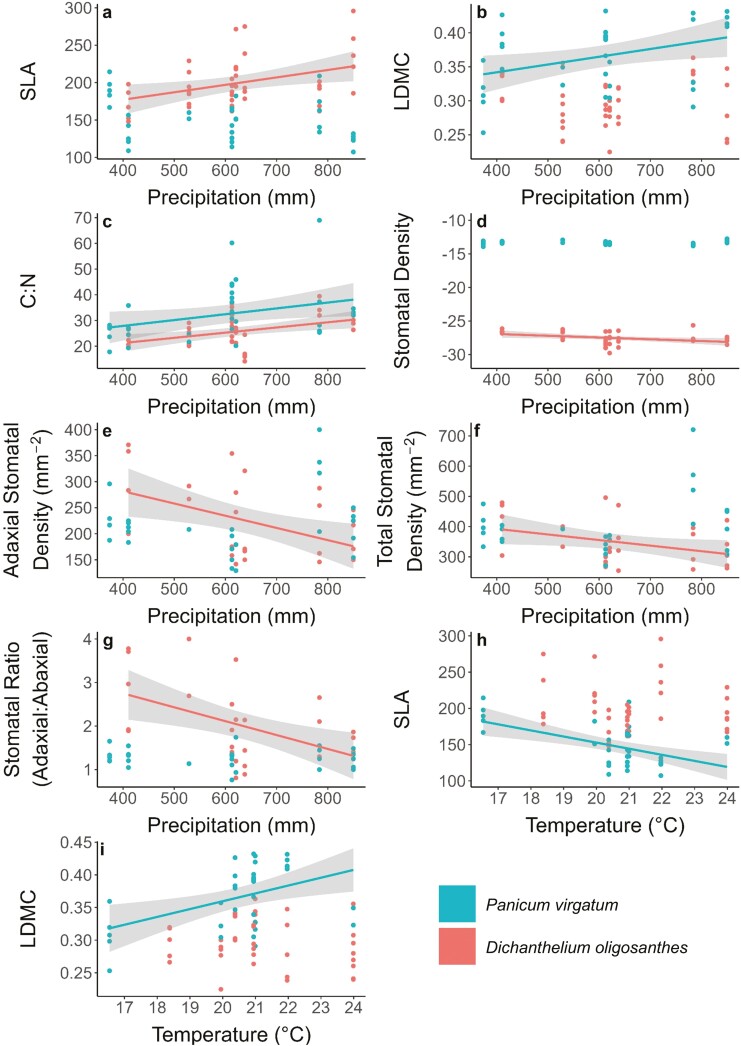
The change of stomatal and structural leaf traits at our grassland sites across precipitation and temperature gradients for *D. oligosanthes* and *P*. *virgatum*. *R*^2^ and *P* values, as well as non-significant results for traits not displayed here can be found in see Supporting Information—Table S3.

Three stomatal traits significantly decreased with increasing precipitation for *D*. *oligosanthes*: adaxial stomatal density (*R*^2^ = 0.23, *P* = 0.009; Fig. 4e), total stomatal density (*R*^2^ = 0.14, *P* < 0.046; Fig. 4f), and adaxial:abaxial stomatal ratio (*R*^2^ = 0.26, *P* = 0.004; Fig. 4g). Stomatal traits did not respond to differences in temperature for *D*. *oligosanthes* and stomatal traits showed no responses to differences in temperature or precipitation for *P*. *virgatum* [**see Supporting Information—Table S3**].

Two structural leaf traits, SLA and C:N, increased with increasing precipitation for *D*. *oligosanthes* (*R*^2^ = 0.15, *P* = 0.013; Fig. 4a and *R*^2^ = 0.20, *P* = 0.003; Fig. 4c, respectively), whereas δ^13^C decreased with increasing precipitation (*R*^2^ = 0.13, *P* = 0.022; Fig. 4d). Leaf traits did not respond to differences in temperature for *D*. *oligosanthes* [**see Supporting Information—Table S3**]. For *P*. *virgatum*, we found that LDMC and C:N both increased with increasing precipitation (*R*^2^ = 0.15, *P* = 0.023; Fig. 4b and *R*^2^ = 0.12, *P* = 0.041; Fig. 4c, respectively). We also found that SLA decreased with increasing temperature (*R*^2^ = 0.31, *P* = 0.001; Fig. 4h) and that LDMC increased with increasing temperature (*R*^2^ = 0.21, *P* = 0.006; Fig. 4i) for *P*. *virgatum*.

## Discussion

Here, we measured a suite of leaf traits on two widespread, closely related grasses representative of the C_3_ (*D. oligosanthes*) and C_4_ (*P. virgatum*) photosynthetic pathways in the Great Plains of North America. We assessed temporal (century-long responses within eastern Kansas) and spatial (across the broader Great Plains of North America) variation in leaf structural and stomatal traits. While we predicted the C_3_ species would be more sensitive to changes in [CO_2_] and climate over time, we found similar temporal responses in C_3_ and C_4_ species as both showed decreased WUE (measured by changes in Δ^13^C) and limited changes in stomatal density in response to increased atmospheric [CO_2_]. Notably, this is the first time a decrease in Δ^13^C has been reported for a C_4_ species. Across the spatial gradient, we found the C_3_ species responded more to the precipitation gradient than the C_4_ species, while the temporal trend identified different traits and relationships for species responses to climate over the past century. These results highlight the intraspecific trait variability that exists according to environmental gradients and changes in [CO_2_], while also clearly illustrating that predictions of spatial trait–climate relationships in the modern record may be unsuitable for predictions of trait–climate relationships over the previous century.

### Trait responses to changes in atmospheric [CO_2_] and climate since 1887

We found limited changes in stomatal density in response to increased atmospheric [CO_2_] or trends in precipitation or temperature over the past 133 years. Our results are contrary to previous studies that have found decreasing stomatal densities in response to elevated [CO_2_] (Peñuelas and Matamala 1990; Beerling and Chaloner 1993a, and 1993b; Woodward and Kelly 1995; Bettarini *et al.* 1998; Doheny-Adams *et al.* 2012; Large *et al.* 2017). Stomatal densities are generally expected to decrease with increased atmospheric [CO_2_] resulting in increased WUE by reducing transpiration. However, atmospheric [CO_2_] is not the only driver of stomatal density, which is genetically determined and sensitive to environmental conditions during leaf maturation (Xu *et al.* 2016). Climate data from within the study region (Manhattan, Kansas, USA) over the last century shows a ~7% increase in mean annual precipitation and 0.93 °C increase in mean annual temperature (Sadayappan *et al.* 2023; Keen *et al.* in press). A progressively wetter and warmer climate in the region over the past century may have limited changes in stomatal density over time as decreased stomatal density and size can constrain gas exchange and limit photosynthesis and leaf cooling via transpiration (Lin *et al.* 2015).

Limited change in stomatal density and size in *D. oligosanthes* may explain increased Δ^13^C over time. The Δ^13^C of *D. oligosanthes* significantly increased with atmospheric [CO_2_] (Fig. 2a), indicating that WUE has decreased through time in this species. Generally, Δ^13^C is expected to decrease in C_3_ species in response to elevated [CO_2_] due to decreased stomatal conductance that reduces water loss without limiting photosynthetic rates (Francey and Farquhar 1982; Peñuelas and Azcón‐Bieto 1992; Araus and Buxo 1993; Pedicino *et al.* 2002). However, other studies have attributed stable Δ^13^C over time to decreases in stomatal density, which maintains *c*_i_/*c*_a_ (ratio of intercellular to atmospheric [CO_2_]) and Δ^13^C under elevated [CO_2_] (Pedicino *et al.* 2002; del Toro *et al.* 2024). We attribute the increase of Δ^13^C in *D*. *oligosanthes* across the studied time period to limited changes in stomatal density corresponding with a century-long trend of increased precipitation in this region.

The negative trend of Δ^13^C over time for *P. virgatum* indicates decreased WUE over the previous century, a response similar to the C_3_ species (Fig. 2b and a, respectively). While few studies have measured temporal changes of Δ^13^C in C_4_ species, only increasing (Pedicino *et al.* 2002; Eastoe and Toolin 2018; del Toro *et al.* 2024) and unchanging (Marino and McElroy 1991; Pedicino *et al.* 2002) trends have previously been reported. To the best of our knowledge, this is the first time a decreasing response of Δ^13^C over time has been reported for a C_4_ species, a finding that may be owed to little research on temporal variation of Δ^13^C in C_4_ plants. The Δ^13^C of C_4_ plants tends to increase when plants are subjected to dry or shady conditions (Buchmann *et al.* 1996; Fravolini *et al.* 2002; Ghannoum *et al.* 2002; Cernusak *et al.* 2013) or increased [CO_2_] over time (del Toro *et al.* 2024). Here, it seems unlikely that the decrease in Δ^13^C over time reflects changes in light conditions, as *P. virgatum* typically grows in full sunlight and these conditions were unchanged over time. Δ^13^C also varies by the subtype of C_4_ photosynthetic pathway (NAD-ME, NADP-ME, and PEP-CK) due to variation in bundle sheath leakiness. The Δ^13^C of the NADP-ME subtype responds the least, followed by PCK then NAD-ME (Buchmann *et al.* 1996). As *P. virgatum* has the NAD-ME subtype, the ~1‰ decrease observed since 1887 is reasonable. Thus, the best explanation for the changes reported here are that increased water availability and temperatures over the past century in the study region (Keen *et al.* in press) may be responsible for the Δ^13^C decrease seen in *P. virgatum*. If precipitation continues to increase in the region, both *P*. *virgatum* and *D*. *oligosanthes* may be expected to continue to reduce their water-use efficiency to maximize growth.

Foliar C:N was positively correlated with atmospheric [CO_2_], suggesting *D. oligosanthes* and *P. virgatum* have responded to CO_2_ fertilization over time (Fig. 2e,f). Plants often increase carbohydrate production more than N uptake under elevated [CO_2_] resulting in nutrient dilution (Peñuelas and Matamala 1990; McLauchlan *et al.* 2010, 2017; Feng *et al.* 2015; Brookshire *et al.* 2020; Peñuelas *et al.* 2020). Changes in C:N ratios in *D. oligosanthes* were also driven by decreased %N and indicate decreased N availability despite increases in anthropogenic N deposition over this time period (McLauchlan *et al.* 2010, 2014). Long-term increases in foliar C:N can limit plant N availability by decreasing foliar decomposition rates and increasing microbial N immobilization (Reich *et al.* 2006; Feng *et al.* 2015). Interestingly, changes in C:N and %N in *D. oligosanthes* showed a stronger relationship with precipitation than [CO_2_], suggesting that directional changes in precipitation may have as large or larger impacts on plant productivity and nutrient dynamics as CO_2_ fertilization. However, the effects of [CO_2_] and precipitation on foliar nutrient concentrations appear to vary according to species (McLauchlan *et al.* 2010) and may reflect species-specific resource requirements (Craine *et al.* 2012) or regional climactic differences (Peñuelas *et al.* 2020). For example, *P. virgatum* is a highly productive species that can displace dominant grasses in areas with high water and N availability (Dybzinski and Tilman 2007; Collins *et al.* 2012; Nieland and Zeglin 2024). As *P. virgatum* has foliar C:N values nearly double that of *D. oligosanthes*, changes in species composition to more productive species would likely have more profound and lasting consequences on ecosystem nutrient dynamics than shifts in species-level C:N alone.

Foliar δ^15^N is often positively correlated with foliar [N] and terrestrial N availability (McLauchlan *et al.* 2010). We found that leaf δ^15^N significantly decreased as atmospheric [CO_2_] increased for both species (Fig. 2g and h), which supported our hypothesis and is consistent with previously described results from grasses, other herbaceous species, and woody species (Peñuelas and Filella 2001; McLauchlan *et al.* 2010; Tang *et al.* 2022). In our study region, plant N availability has decreased despite increased anthropogenic N deposition since at least the 1980s (McLauchlan *et al.* 2014). Changes in foliar δ^15^N over time may reflect increased N limitation as described by the progressive nitrogen limitation hypothesis—the idea that increased atmospheric [CO_2_] causes nitrogen to become more limited in the soil due to increased N immobilization and sequestration of N by plants benefitting from elevated photosynthetic rates (Luo *et al.* 2004). Alternatively, changes in foliar δ^15^N may reflect changes in the isotopic signature of N taken up by the plant rather than changes in N availability (Tang *et al.* 2022). Foliar depletion of δ^15^N may be an artefact of changes to the ecosystem N signature due to N deposition (Tang *et al.* 2022). Decreased δ^15^N may also reflect increased mycorrhizal activity under elevated [CO_2_] as mycorrhizae tend to deliver depleted N to plants (McLauchlan *et al.* 2010; Hobbie and Högberg 2012). While we cannot determine the mechanisms driving decreased δ^15^N in this study, it is likely this often-reported response is due to a combination of N limitation and altered N signature under environmental change.

### Trait variation across the Great Plains

We measured a suite of traits on individuals of *D. oligosanthes* and *P. virgatum* across eight grassland sites located throughout the Great Plains of North America. We predicted that we would find differences within stomatal and structural leaf traits across precipitation and temperature gradients for both grasses, as these species persist across a wide range of environments with complex temporal and spatial variability.

Species may exhibit intraspecific variation of structural leaf traits to gain competitive advantages across environmental conditions (Reich 2014) or across climate gradients (Griffin-Nolan *et al.* 2018). As discussed by Griffin-Nolan and Sandel (2023), inconsistent trait responses by grass species to mean climate conditions can reflect a variety of other biotic and abiotic factors, including soil characteristics, local topography, and canopy cover. Here, we reported several leaf-traits correlated with the axes of climate variability, and a few traits that did not vary by climate. For instance, we found that *P*. *virgatum* decreased SLA and increased LDMC as temperature increased, whereas leaves of *D*. *oligosanthes* showed no differences (Fig. 4h and i). In contrast, leaves of *D*. *oligosanthes* had greater SLA at wetter sites, favouring more rapid growth with increased water availability, whereas the leaves of *P*. *virgatum* did not change significantly (Fig. 4a). We found that *D*. *oligosanthes* and *P*. *virgatum* shared only one similar significant result across all leaf traits we measured: C:N (Fig. 4c). The increase in C:N across greater precipitation is likely due to growth dilution of N, where both grass species accrue more carbon in wetter environments and consequently dilute the abundance of N in leaves with greater area.

Though stomatal densities and sizes have been found to change across precipitation and temperature gradients (Pyakurel and Wang 2014; Hill *et al.* 2015; Carlson *et al.* 2016; Du *et al.* 2021), few differences were found for the species investigated here. Stomatal traits remained unchanged for *P*. *virgatum* across gradients that differ in mean precipitation or temperature (Table S3). For *D*. *oligosanthes*, we found that adaxial and total stomatal density and stomatal ratio decreased with increasing precipitation (Fig. 4e–g). This decrease in stomatal density is likely an artefact of increasing SLA and leaf size with increasing precipitation (Fig. 4a); if the number of stomata per leaf remains constant as leaf area increases, then stomatal density will decrease.

Lastly, we also observed a decrease in δ^13^C with increasing precipitation in *D*. *oligosanthes* (Fig. 4d), a trend that was previously observed in C_3_ grasses across a precipitation gradient (Weiguo *et al.* 2005). In C_3_ plants, differences in δ^13^C are strongly driven by instantaneous *c*_i_/*c*_a_, the ratio of intracellular [CO_2_] to the ratio of atmospheric [CO_2_] (Cernusak *et al.* 2013). Instantaneous *c*_i_/*c*_a_ has a negative relationship with leaf δ^13^C (Cernusak *et al.* 2013) and is influenced by many environmental factors including water availability, nutrient availability, irradiance, and reduced CO_2_ partial pressures due to elevation (Tieszen 1991). Tieszen (1991) predicted irradiance affects *c*_i_/*c*_a_ the most and water availability second, but *D*. *oligosanthes* was collected in open grasslands at all sites in this study, so differences in irradiance are likely unimportant as drivers of δ^13^C in this dataset. Reduced water availability decreases *c*_i_/*c*_a_ by increasing stomatal regulation and decreasing discrimination against ^13^C, resulting in higher foliar δ^13^C values (Tieszen 1991; Cernusak *et al.* 2013). We conclude that it is unlikely that other factors besides differences in growing season precipitation across sites are driving this trend of decreasing δ^13^C across the precipitation gradient of the Great Plains.

## Conclusion

Using a long temporal record and an extensive spatial record, we reported both similarities and unique responses to varying environmental conditions in two closely related grass species, *D. oligosanthes* (C_3_) and *P. virgatum* (C_4_). Using herbarium samples, leaf Δ^13^C suggested that both species reduced water-use efficiency in response to century-long increases in water availability. For some traits, such as stomatal density, hypothesized responses to environmental changes over the past century were not evident, which contrasts with results from other studies. When the same leaf traits were measured in field populations sampled across precipitation and temperature gradients in the Great Plains, we found that many traits, including SLA, LDMC, C:N, δ^13^C, adaxial stomatal density, total stomatal density, and stomatal ratio had statistically-significant relationships with spatial patterns of precipitation, while fewer traits (SLA and LDMC for *P*. *virgatum* only) had statistically-significant responses to spatial variation in temperature. These results demonstrate the importance of characterizing trait variation across both temporal and spatial scales. For instance, predictions of how C_3_ and C_4_ grasslands will change in the future are usually made based on the examination of the results of just a few grass species in the modern record. Our work shows that many typically held assumptions of how traits will change in response to environmental variables vary with different trait–climate relationships across space and time. If we are to understand how plants will respond to global change, especially with regard to changes in precipitation regimes, it may be necessary to first document how plants have responded to historical changes in the environment, as well as the intraspecific trait variation that currently exists.

## Supporting Information

The following additional information is available in the online version of this article –

**Table S1:** A list of herbarium specimens sampled at KSC and KANU.

**Table S2:** Study sites and their mean 30-year growing season precipitation and temperature.

**Table S3:**  *R*^2^ and *P* for all traits measured across precipitation and temperature gradients at the grassland sites.

**Fig. S1:** Graphs of stomatal leaf traits that responded significantly to changes in growing season precipitation and temperature as measured across the years 1887–2020.

**Fig. S2:** Graphs of non-stomatal leaf traits that responded significantly to changes in growing season precipitation and temperature as measured across the years 1887–2020.

plae055_suppl_Supplementary_Tables_S1-S3_Figures_S1-S2

## Data Availability

Trait data collected for this project and the associated R code are available from the Konza Prairie LTER Data Catalog http://lter.konza.ksu.edu/data (doi: 10.6073/pasta/dbbb04ad458ea29bbb94a40c6f4f13ba).
