# Correction to: Grass leaf structural and stomatal trait responses to climate gradients assessed over the 20th century and across the Great Plains, USA

**DOI:** 10.1093/aobpla/plae064

**Published:** 2024-12-09

This is a correction to: Ryan C Donnelly, Jesse B Nippert, Emily R Wedel, Carolyn J Ferguson, Grass leaf structural and stomatal trait responses to climate gradients assessed over the 20th century and across the Great Plains, USA, *AoB PLANTS*, Volume 16, Issue 5, October 2024, plae055, https://doi.org/10.1093/aobpla/plae055

In the originally published version of the manuscript, there was an error in a name within the Acknowledgements section. “Jennifer Jewitt” should read: “Jennifer Jewett”.

The emendation has been made to the article.

