# Supplementary material for: Grass leaf structural and stomatal trait responses to climate gradients assessed over the 20th century and across the Great Plains, USA
Source: AoB Plants. 2024 Sep 26;16(5):plae055. doi: 10.1093/aobpla/plae055 (PMC11489733; doi:10.1093/aobpla/plae055)
Supplement: plae055_suppl_Supplementary_Tables_S1-S3_Figures_S1-S2 [file plae055_suppl_supplementary_tables_s1-s3_figures_s1-s2.pdf]

***AoB PLANTS* Supporting Information**

Article title: Grass leaf structural and stomatal trait responses to climate gradients assessed over the 20th century and across the Great Plains, USA

The following Supporting Information is available for this article:

Table S1: A list of herbarium specimens sampled at Kansas State University Herbarium (KSC) and the Ronald L. McGregor Herbarium at the University of Kansas (KANU).

Species	Date	County	Herbarium	Barcode ID
Dichanthelium oligosanthes	June 1887	Riley	KSC	14877
Dichanthelium oligosanthes	May 28, 1890	Pottawatomie	KSC	14909
Dichanthelium oligosanthes	June 28, 1896	Wyandotte	KSC	14892
Dichanthelium oligosanthes	June 23, 1921	Riley	KSC	14872
Dichanthelium oligosanthes	May 30, 1928	Sumner	KSC	14866
Dichanthelium oligosanthes	May 24, 1935	Saline	KSC	14915
Dichanthelium oligosanthes	June 13, 1936	Sumner	KSC	14822
Dichanthelium oligosanthes	June 25, 1957	Doniphan	KSC	14918
Dichanthelium oligosanthes	June 9, 1960	Woodson	KSC	14812
Dichanthelium oligosanthes	June 10, 1967	Cowley	KSC	14801
Dichanthelium oligosanthes	July 14, 1977	Riley	KSC	1353
Dichanthelium oligosanthes	June 8, 1987	Riley	KSC	1354
Dichanthelium oligosanthes	May 25, 1998	Saline	KSC	14923
Dichanthelium oligosanthes	June 18, 2008	Washington	KSC	127646
Dichanthelium scribnerianum	June 8, 1950	Franklin	KANU	13960
Dichanthelium scribnerianum	May 14, 1955	Anderson	KANU	13864
Dichanthelium scribnerianum	May 27, 1959	Franklin	KANU	13891
Dichanthelium scribnerianum	May 11, 1963	Woodson	KANU	13925
Dichanthelium scribnerianum	June 7, 1966	Chase	KANU	13839
Dichanthelium scribnerianum	June 15, 1971	Ottawa	KANU	13985

Dichanthelium scribnerianum	June 16, 1971	Cloud	KANU	13930
Dichanthelium scribnerianum	June 2, 1975	Comanche	KANU	13923
Dichanthelium scribnerianum	June 24, 1975	Elk	KANU	13924
Dichanthelium scribnerianum	June 6, 1988	Marshall	KANU	72290
Dichanthelium scribnerianum	June 13, 1990	Douglas	KANU	105263
Dichanthelium scribnerianum	June 25, 1993	Johnson	KANU	109388
Dichanthelium scribnerianum	June 11, 1994	Miami	KANU	115118
Dichanthelium scribnerianum	June 12, 1999	Osage	KANU	322622
Dichanthelium scribnerianum	June 18, 2002	Woodson	KANU	342388
Dichanthelium scribnerianum	May 26, 2003	Douglas	KANU	341731
Dichanthelium scribnerianum	May 24, 2013	Cherokee	KANU	421634
Panicum virgatum	July 22, 1949	Shawnee	KANU	19408
Panicum virgatum	August 10, 1955	Labette	KANU	19418
Panicum virgatum	August 7, 1958	Barber	KANU	19373
Panicum virgatum	July 8, 1961	Morton	KANU	19567
Panicum virgatum	August 10, 1963	Woodson	KANU	19339
Panicum virgatum	July 22, 1969	Rice	KANU	19405
Panicum virgatum	July 23, 1972	Kiowa	KANU	19555
Panicum virgatum	July 19, 1976	Edwards	KANU	19395
Panicum virgatum	August 15, 1983	Sherman	KANU	19479
Panicum virgatum	July 17, 1985	Barton	KANU	19297
Panicum virgatum	August 8, 1989	Jefferson	KANU	105459
Panicum virgatum	August 6, 1992	Douglas	KANU	107348
Panicum virgatum	August 31, 1998	Cheyenne	KANU	320201
Panicum virgatum	July 18, 1999	Reno	KANU	326227
Panicum virgatum	July 11, 2002	Riley	KANU	339150
Panicum virgatum	August 16, 2003	Saline	KANU	343108
Panicum virgatum	June 25, 2013	Labette	KANU	421694
Panicum virgatum	July 26, 1887	Sumner	KSC	32290
Panicum virgatum	July 1895	Ottawa	KSC	32356

Panicum virgatum	July 29, 1897	Brown	KSC	32324
Panicum virgatum	August 22, 1927	Riley	KSC	32347
Panicum virgatum	August 14, 1935	Saline	KSC	32237
Panicum virgatum	August 10, 1937	Cloud	KSC	32314
Panicum virgatum	August 26, 1953	Sedgwick	KSC	32317
Panicum virgatum	July 28, 1962	Riley	KSC	32309
Panicum virgatum	July 7, 1967	Cowley	KSC	32210
Panicum virgatum	July 8, 1977	Riley	KSC	1455
Panicum virgatum	July 12, 1979	Riley	KSC	1457
Panicum virgatum	July 27, 1994	Geary	KSC	32227
Panicum virgatum	June 25, 1995	Neosho	KSC	32220
Panicum virgatum	August 24, 2007	Washington	KSC	106933

Table S2: A list of our study sites and their mean 30-year growing season precipitation and temperature used to calculate results.

Site	Mean 30-Year Growing Season Precipitation (mm)	Mean 30-Year Growing Season Temperature (°C)	Latitude and Longitude
Woodworth Station Waterfowl Production Area, North Dakota	374	16.5	47.138147, -99.242352
Cedar Creek Ecosystem Science Reserve, Minnesota	529	24	45.401846, -93.200305
Valentine National Wildlife Refuge, Nebraska	410	20.4	42.486730, -100.520071
T. L. Davis Preserve, Nebraska	621	19.9	41.197590, -96.298930
Kish-Ke-Kosh Prairie, Iowa	638	18.4	41.565294, -93.013477
Konza Prairie Biological Station, Kansas	613	20.9	39.107101, -96.609108
Wah'Kon-Tah Prairie, Missouri	784	21	37.905991, -93.992905
Joseph H. Williams Tallgrass Prairie Preserve, Oklahoma	850	22	36.846530, -96.422883

Table S3: R^2 and P for all traits measured at our grassland sites across precipitation and temperature gradients for both *D. oligosanthes* and *P. virgatum*. Bolding indicates significance ($P < 0.05$).

Species	Environmental Variable	Trait	R^2	*P*
Dichanthelium oligosanthes ssp. *scribnerianum*	Mean 30-Year Growing Season Precipitation (mm)	SLA	0.15	**0.013**
		LDMC	0.00	0.798
		C:N	0.20	**0.003**
		$\delta^{13}C$	0.13	**0.022**
		Leaf Thickness	0.00	0.73
		Stomatal Density (Abaxial)	0.06	0.210
		Stomatal Density (Adaxial)	0.23	**0.009**
		Stomatal Length (Abaxial)	0.01	0.691
		Stomatal Length (Adaxial)	0.07	0.164
		Stomatal Density (Whole Leaf)	0.14	**0.046**
		Stomatal Ratio (Adaxial:Abaxial)	0.26	**0.004**
	Mean 30-Year Growing Season Temperature (°C)	SLA	0.00	0.817
		LDMC	0.03	0.295
		C:N	0.07	0.095
		$\delta^{13}C$	0.07	0.095
		Leaf Thickness	0.04	0.240
		Stomatal Density (Abaxial)	0.13	0.051
		Stomatal Density (Adaxial)	0.01	0.688
		Stomatal Length (Abaxial)	0.10	0.090
		Stomatal Length (Adaxial)	0.01	0.855
		Stomatal Density (Whole Leaf)	0.01	0.6939
		Stomatal Ratio (Adaxial:Abaxial)	0.07	0.162
Panicum virgatum	Mean 30-Year Growing Season Precipitation (mm)	SLA	0.10	0.0673
		LDMC	0.15	**0.023**
		C:N	0.12	**0.041**
		$\delta^{13}C$	0.00	0.927
		Leaf Thickness	0.01	0.494
		Stomatal Density (Abaxial)	0.10	0.104
		Stomatal Density (Adaxial)	0.03	0.367
		Stomatal Length (Abaxial)	0.03	0.392
		Stomatal Length (Adaxial)	0.10	0.104
		Stomatal Density (Whole Leaf)	0.07	0.195
		Stomatal Ratio (Adaxial:Abaxial)	0.01	0.597
	Mean 30-Year Growing Season Temperature (°C)	SLA	0.31	**0.001**
		LDMC	0.21	**0.006**
		C:N	0.04	0.263
		$\delta^{13}C$	0.07	0.136
		Leaf Thickness	0.03	0.503

		Stomatal Density (Abaxial)	0.01	0.688
		Stomatal Density (Adaxial)	0.00	0.818
		Stomatal Length (Abaxial)	0.00	0.931
		Stomatal Length (Adaxial)	0.00	0.946
		Stomatal Density (Whole Leaf)	0.00	0.982
		Stomatal Ratio (Adaxial:Abaxial)	0.04	0.303

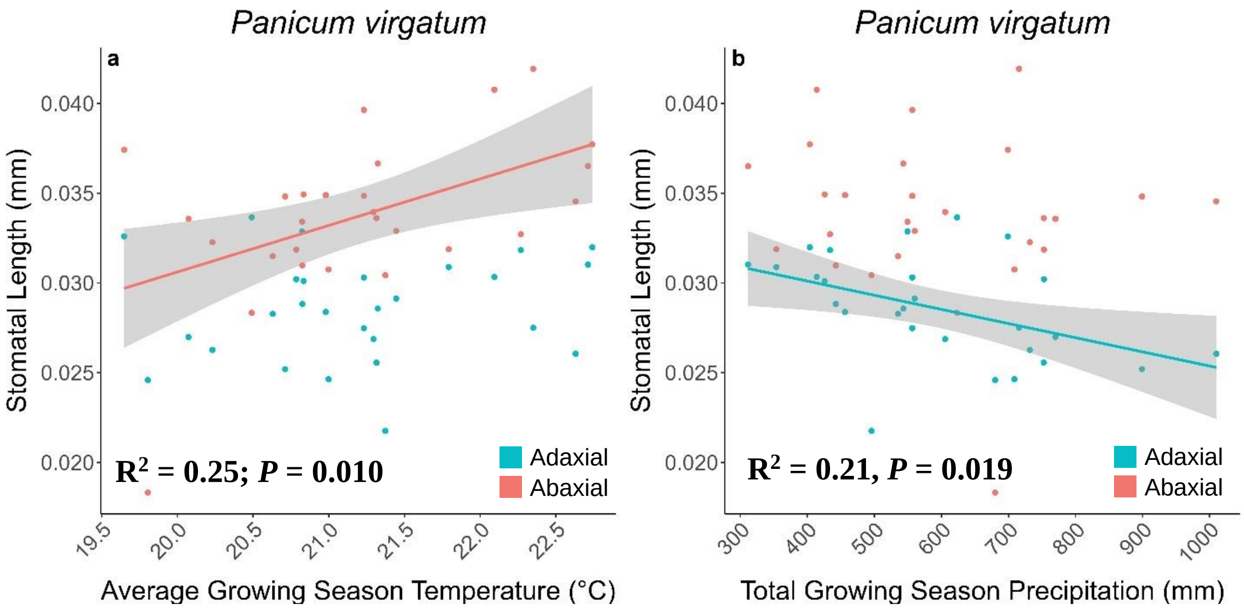

Fig S1: The change in stomatal length of *P*. *virgatum* with temperature (left column) and precipitation (right column) as measured across the years 1887 – 2020. Regression lines and confidence intervals are displayed when $P < 0.05$.

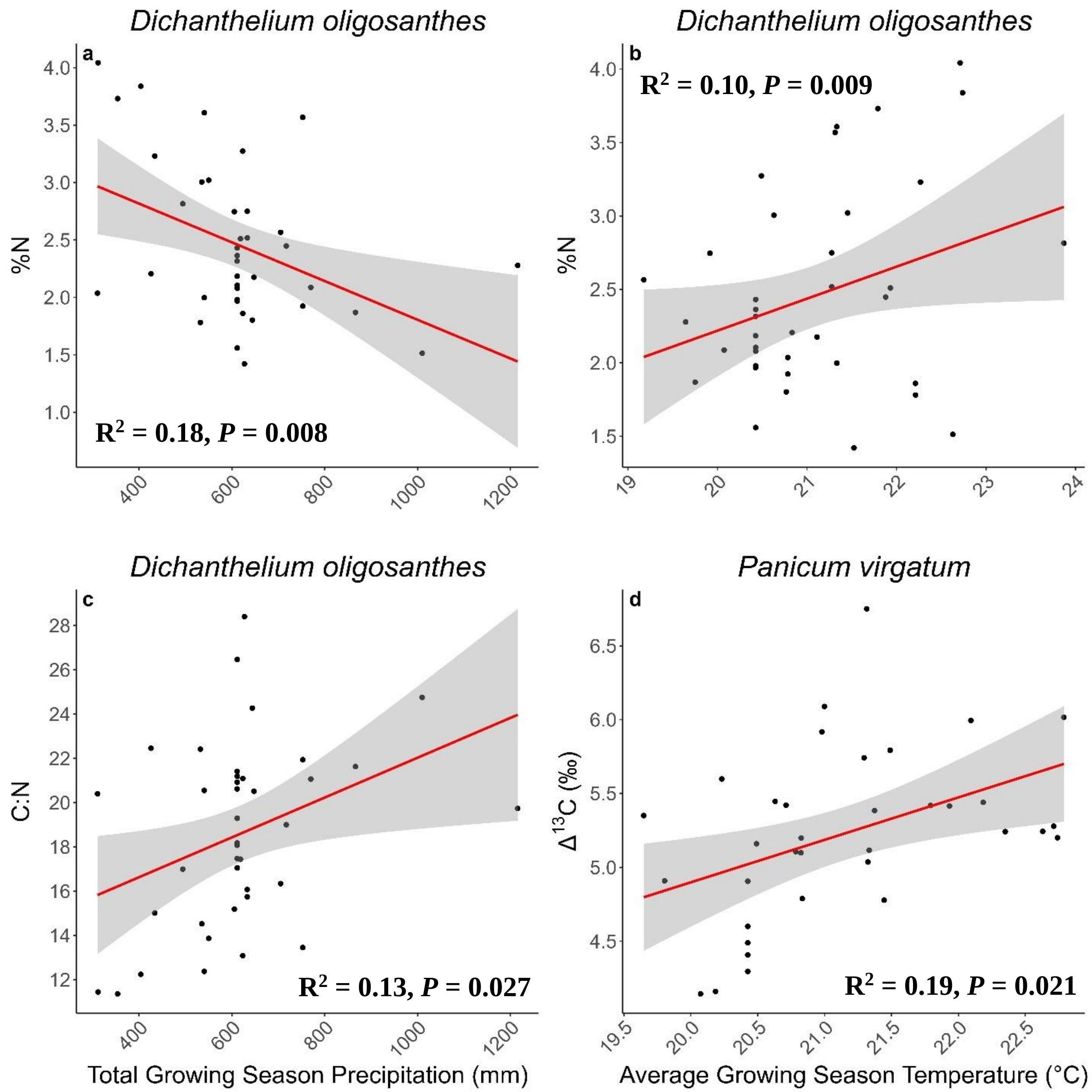

Fig S2: The change in leaf traits of *P. virgatum* and *D. oligosanthes* with precipitation (left column) and temperature (right column) as measured across the years 1887 – 2020. Regression lines and confidence intervals are displayed when $P < 0.05$.
